# Asymmetric Bilateral Medial Temporal Lobe Encephalitis Associated With Anti-N-Methyl-D-Aspartate (NMDA) Receptor Antibodies: A Case Report

**DOI:** 10.7759/cureus.74255

**Published:** 2024-11-22

**Authors:** Iram Fatima, Dhruv Kundu, Ahmed Sadain Khalid, Sara Tabassum, Vishwesh Patel, Shreya Muralidharan, Yusra Qamar

**Affiliations:** 1 Internal Medicine, Holy Name Medical Center, Teaneck, USA; 2 General Medicine, Peterborough City Hospital, Peterborough, GBR; 3 Internal Medicine, Faisalabad Medical University, Faisalabad, PAK; 4 Psychiatry, Dr VRK Women’s Medical College, Hyderabad, IND; 5 Internal Medicine, Shri Meghji Pethraj (MP) Shah Government Medical College, Jamnagar, IND; 6 Internal Medicine, Vydehi Institute of Medical Sciences and Research Centre, Bangalore, IND; 7 Surgery, Lala Lajpat Rai Hospital, Kanpur, IND

**Keywords:** anti-nmda receptor encephalitis, asymmetric involvement, atypical presentation, neuropsychiatric symptoms, ovarian teratoma

## Abstract

Anti-N-methyl-D-aspartate (NMDA) receptor encephalitis is a rare autoimmune disorder that typically presents with neuropsychiatric symptoms and medial temporal lobe involvement. We report the case of a 24-year-old female with no significant medical history, who developed severe anxiety, memory deficits, and confusion over a two-week period. Neurological examination revealed cognitive dysfunction, asymmetric limb movements, and psychosis. MRI showed bilateral medial temporal lobe hyperintensities, more pronounced on the left, and CSF analysis confirmed elevated protein levels and positive anti-NMDA receptor antibodies. Treatment with high-dose corticosteroids and intravenous immunoglobulin led to significant improvement within three weeks, with a follow-up MRI showing a reduction in lesion size. This case underscores the importance of early diagnosis, particularly when atypical presentations, such as asymmetric temporal lobe involvement, are observed. Awareness of this condition, especially in young women, is critical for timely intervention and management, given its potential association with ovarian teratomas.

## Introduction

Encephalitis refers to brain inflammation with various potential causes, with infections being the most common. However, in the last decade, noninfectious encephalitis, particularly autoimmune forms, has been on the rise, posing a significant diagnostic challenge. Several cases of autoimmune encephalitis are associated with malignancies, especially ovarian teratomas in females [1,2]. Anti-N-methyl-D-aspartate (NMDA) receptor encephalitis is an autoimmune disorder characterized by neuropsychiatric symptoms, seizures, and abnormal movements. This condition is mediated by antibodies that target the NMDA receptor 1 subunit of NMDA receptors, disrupting neurotransmission. NMDA receptors, primarily located in the hippocampus and prefrontal cortex, are essential for synaptic plasticity, learning, and memory, and play a role in synapse formation and stabilization during brain development [1].

NMDA receptor encephalitis typically begins with a prodromal phase of flu-like symptoms, including fever, fatigue, and headache, which lasts for days to weeks. This is followed by an acute phase characterized by psychiatric symptoms such as confusion, hallucinations, and delusions [3,4]. These early psychiatric symptoms may mimic schizophrenia or acute psychosis, leading to diagnostic delays. Patients may rapidly deteriorate, developing movement disorders (such as chorea or dystonia), catatonia, and altered sensorium. Autonomic dysfunction, hypotension, and bradycardia may also occur, potentially being life-threatening [5]. Ovarian teratomas often contain neural tissue, and antibodies produced against the tumor can cross-react with NMDA receptors, resulting in neurological symptoms. These cases typically show poor responses to immunotherapy but may improve with chemotherapy [6].

Asymmetric involvement of the medial temporal lobes is an atypical manifestation of autoimmune encephalitis, often affecting the hippocampus, which is crucial for memory and emotional regulation. Key symptoms include short-term memory loss, confusion, seizures, and emotional lability [7]. Asymmetric involvement may resemble viral or paraneoplastic encephalitis, complicating diagnosis. Therefore, distinguishing between infectious and autoimmune causes of encephalitis is essential. Diagnosis of NMDA receptor encephalitis is confirmed by detecting anti-NMDA receptor antibodies in the CSF. CSF analysis may also show elevated white blood cell count and protein levels. Although antibodies can be detected in the serum, CSF testing is more sensitive. In the early stages, MRI may appear normal, although some patients show T2-weighted imaging/fluid-attenuated inversion recovery (FLAIR) hyperintensities in the medial temporal lobes, indicative of limbic encephalitis. This involvement may be symmetric or, as in the present case, asymmetric [8]. We present the case of a 24-year-old female diagnosed with asymmetric bilateral medial temporal lobe encephalitis, confirmed to be anti-NMDA receptor encephalitis.

## Case presentation

A 24-year-old female with no significant past medical history presented to the emergency department with severe anxiety, hallucinations, and memory deficits that had developed over the previous two weeks. Her condition was characterized by marked confusion, including an inability to recognize family members. This combination of symptoms prompted her family to seek urgent medical attention.

Upon neurological examination, the patient exhibited cognitive dysfunction, including impaired short-term memory and disorientation to time and place. She displayed neurological signs such as asymmetric limb movements and right-sided hyperreflexia. Her behavior was notably agitated, with episodes of psychosis complicating her clinical picture. These findings raised concerns about underlying neurological or psychiatric conditions.

**Figure 1:**
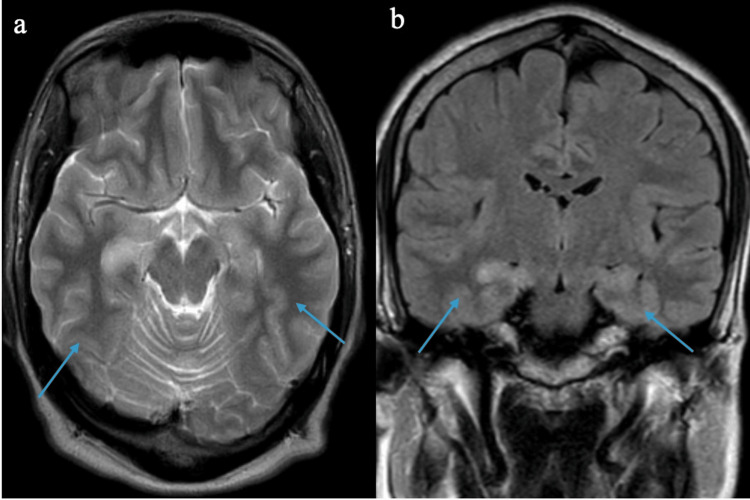
MRI showing bilateral medial temporal lobe abnormalities with hyperintensities

Diagnostic investigations were promptly initiated. MRI of the brain revealed bilateral medial temporal lobe abnormalities, more pronounced on the left side, with hyperintensities suggestive of inflammation (Figure 1). CSF analysis showed elevated protein levels and lymphocytic pleocytosis, while cultures and PCR tests for common infectious agents returned negative (Table 1). Importantly, serological testing confirmed the presence of anti-NMDA receptor antibodies, guiding the clinical diagnosis toward autoimmune encephalitis. Differential diagnoses considered included viral encephalitis, neurodegenerative disorders, and psychiatric disorders.

**Table 1:** CSF and diagnostic findings in anti-NMDA receptor encephalitis NMDA, N-methyl-D-aspartate

Diagnostic test	Result	Reference range
CSF protein	100 mg/dL	15-45 mg/dL
CSF cell count	30 cells/µL	0-5 cells/µL
CSF lymphocyte count	70% lymphocytes	0-5% lymphocytes
Culture and PCR for infectious agents	Negative	Negative for common pathogens (e.g., bacteria and viruses)
Serological testing (anti-NMDA antibodies)	Positive (1:1,000 titer)	Negative

Management was initiated with high-dose corticosteroids (methylprednisolone), followed by intravenous immunoglobulin therapy. In addition to pharmacological treatment, supportive care was provided, focusing on psychiatric management for her agitation and cognitive rehabilitation to address her cognitive deficits. After three weeks of treatment, the patient exhibited marked improvement in cognitive function and behavioral stability. Follow-up MRI showed a reduction in the size of the lesions, indicating a positive response to therapy. She continued outpatient therapy, including cognitive rehabilitation, with ongoing monitoring for any residual neurological deficits, which suggested a hopeful prognosis for her recovery.

## Discussion

Anti-NMDA receptor encephalitis is a rare autoimmune paraneoplastic disorder predominantly affecting children as young as two months and young adults, with fewer than 5% of cases occurring in individuals over 45 years old. The condition is more common in females, with a male-to-female ratio of 1:4, and is frequently associated with ovarian teratomas [9].

The diagnosis is confirmed by detecting IgG antibodies targeting the GluN1 subunit of NMDA receptors in both serum and CSF. However, these tests may not be readily available during initial presentations in the emergency department. Therefore, when a patient presents with a suggestive clinical picture, a lumbar puncture should be performed to assess for CSF pleocytosis or oligoclonal bands. EEG is also useful, as it frequently reveals slow and disorganized epileptic activity, although these findings are nonspecific [10,11]. In our patient, CSF analysis showed elevated protein levels and lymphocytic pleocytosis.

Diagnosing anti-NMDA receptor encephalitis can be challenging due to its diverse clinical presentation. However, several key features can aid in identification. These include young women, typically in their second to fifth decades of life; psychiatric manifestations that present unusually; MRI findings that may be normal or atypical; and the presence of benign ovarian tumors in some cases. In our patient, the presentation included severe anxiety, hallucinations, and memory deficits, although no significant oncological history was noted.

Understanding MRI findings is critical for diagnosing anti-NMDA receptor encephalitis. In a case series of 100 patients studied by Dalmau et al., MRI abnormalities were identified in 55 patients, with enhanced signaling on FLAIR or T2 sequences [1]. Among these, 14 patients showed transient contrast enhancement of the cerebral cortex, meninges, or basal ganglia. There were 19 patients with involvement of a single brain area, with 16 exhibiting abnormalities in the medial temporal lobe, two in the corpus callosum, and one in the brainstem, as summarized in Table 2 [1].

**Table 2:** MRI abnormalities in anti-NMDA receptor encephalitis NMDA, N-methyl-D-aspartate Source: Hartung et al. (2024) [12]

Lesion location	Number of patients
Medial temporal lobes	22
Cerebral cortex	17
Cerebellum	6
Brainstem	6
Basal ganglia	5
Contrast enhancement in cortex, meninges, and basal ganglia	14
Other (corpus callosum, hypothalamus, periventricular, and white matter)	8

The varied MRI findings in anti-NMDA receptor encephalitis emphasize the importance of recognizing atypical presentations, as highlighted by our case of asymmetric medial temporal lobe involvement. Although bilateral involvement is more common due to the pathogenesis involving autoantibodies, it is essential to remain aware of less typical patterns to ensure timely intervention and optimal patient outcomes [12].

For instance, the literature includes a case of a 24-year-old female who initially exhibited symptoms resembling a urinary tract infection and atypical pneumonia. She was only diagnosed with autoimmune encephalitis upon developing neuropsychiatric symptoms, prompting further laboratory investigations, including CSF analysis, serology, and imaging [13]. Another case involved a 13-year-old whose symptoms mimicked Guillain-Barré syndrome, delaying the diagnosis of anti-NMDA receptor encephalitis due to the absence of typical psychiatric manifestations [14].

Diagnostic tests, such as CSF analysis showing elevated protein levels and lymphocytic pleocytosis, as well as positive serology for anti-NMDA receptor antibodies, demonstrate high variability in detection. Some cases may test positive for autoantibodies only at later stages of the disease [15]. In contrast, there are cases where early detection of anti-NMDA receptor antibodies correlates with the onset of psychiatric symptoms. In one such case, the relationship between anti-NMDA receptor encephalitis and ovarian teratoma was established when the teratoma was discovered during follow-up, despite not being evident during the initial presentation of encephalitis [16].

Additionally, some patients with severe psychiatric symptoms, including suicidal ideation and agitation, initially show negative results in imaging, serology, and CSF tests. However, EEG findings, such as extreme delta brush waves, suggest encephalopathy and prompted the diagnosis of anti-NMDA receptor encephalitis, ultimately revealing a right adnexal mass consistent with an ovarian teratoma [17].

Given the wide range of presentations, it is crucial to consider a thorough diagnostic approach, including detailed CSF analysis, serologic testing, imaging studies, and a careful search for associated tumors when evaluating patients with neuropsychiatric symptoms suggestive of anti-NMDA receptor encephalitis.

## Conclusions

This case report underscores the importance of recognizing atypical presentations of anti-NMDA receptor encephalitis, particularly asymmetric medial temporal lobe involvement. Severe neuropsychiatric symptoms and cognitive dysfunction can mimic other conditions, resulting in diagnostic delays. Timely imaging, CSF analysis, and serological testing are crucial for effective management. The patient’s significant improvement with immunotherapy highlights the importance of prompt intervention, especially in young women. Ongoing research and education on the variability of this condition are essential to improve patient outcomes.
